# Patient-related factors influencing the choice of haemodialysis access in Sweden

**DOI:** 10.1177/11297298251357632

**Published:** 2025-07-23

**Authors:** Emelie Laveborn, Ellinor Bergdahl, Ulrika Hahn Lundström, Bernd Stegmayr, Michael Ott

**Affiliations:** 1 Department of Public Health and Clinical Medicine, Umeå University, Umeå, Sweden; 2 Division of Renal Medicine, Department of Clinical Science, Intervention and Technology, Karolinska Institutet, Stockholm, Sweden

**Keywords:** Dialysis access, AV fistula, catheters, prosthetic grafts, upper-arm fistula

## Abstract

**Background:**

Haemodialysis access patterns differ internationally. This can not only be explained by differences in patient cohorts. What is considered *the right access for the right patient* is debated and it is unclear which patient-related factors affect the choice of access. The aim of the study was to investigate how patient-specific factors as body size and comorbidities influenced the choice of haemodialysis access in a real-life setting.

**Methods:**

Retrospective cohort study including all patients receiving a haemodialysis access in Sweden between 2013 and 2022. Data from the Swedish Renal Registry (SNR) and the National Patient Register (NPR) was used. Data regarding age, sex, cause of kidney failure, previous kidney replacement therapy, height and weight (after dialysis), were collected from SNR. Data on comorbidities were extracted both from SNR and the NPR. AV-accesses were grouped into four categories depending on location of artery. Changes in arteriovenous access creation over time and patient-related factors affecting the choice of first access were analysed.

**Results:**

Of 10,170 patients, 9706 with 17,709 accesses were included. The creation of upper-arm fistulas (*p* = 0.042) and arteriovenous grafts (*p* = 0.007) increased. Small body size, female sex, diabetes mellitus, vintage, previous haemodialysis treatment (all *p* < 0.001), age (*p* = 0.002) and peripheral arterial disease (*p* = 0.031) led to more central venous catheters. Small body size, female sex, peripheral arterial disease, vintage, previous haemodialysis treatment (all *p* < 0.001) and diabetes mellitus (*p* = 0.023) decreased the probability for selecting a forearm fistula. Upper-arm fistulas were preferred over arteriovenous grafts for those with small body size (*p* < 0.001 for body surface area), female sex (*p* = 0.003) and previous haemodialysis (*p* < 0.001).

**Conclusions:**

The use of upper-arm fistulas and arteriovenous grafts is increasing, while forearm arteriovenous fistulas remain the primary access modality. Patient-related factors influencing the choice of access seemed to be related to vessel size and quality, rather than age and cardiovascular comorbidities.

## Introduction

The haemodialysis access is the lifeline for every patient on haemodialysis. International guidelines advocate forearm arteriovenous fistula (forearm-AVF) as the primary choice due to a lower risk of complications.^1,2^ Central vein catheters (CVC) have predominantly been linked to a high risk of infections, leaving them as a last hand choice. Ultimately, the decision of which access to create should be based on the clinician’s ‘best judgement’.^1^

Different accesses have different advantages and disadvantages. On one hand, there are indications that AV-fistulas may increase the risk of heart failure.^3–5^ This risk might be more pronounced in the presence of an upper-arm fistula (UAF). Compared to a forearm-AVF, UAF have an increased risk of higher access blood flow (Qa). Eventually, this may cause high output heart failure.^6,7^ On the other hand, forearm-AVF have a lower primary patency and a higher need for repeated interventions than UAF or arteriovenous grafts (AVG), especially in the elderly.^8,9^ Therefore, UAF or AVG have been proposed as the primary choice of access in this group.^10,11^ AVG are linked to increased risk of all cause and infection-related hospitalization.^12^ AVG also carry an increased mortality risk.^13^ There is conflicting evidence whether AVG have lower patency compared to UAF.^14–18^

Results from the Dialysis Outcomes and Practice Patterns Study (DOPPS) showed an increase in UAF use between 1996 and 2015.^19^ This may be a result of the *fistula first initiative*^20^ that aimed to actively decrease the use of AVG.

Previous studies have often focused on patency and complications, but little is known about which factors affect the choice of access for the individual patient. In 2019, KDOQI (Kidney Disease Outcomes Quality Initiative) updated its recommendations, replacing ‘fistula first’ with a more nuanced and patient-centred approach.^1,21^ From available data, it is hard to differentiate if UAF replace CVC and AVG in adherence to the *fistula first initiative*^20^ or if UAF are preferred over forearm-AVF in patients where data indicate that maturation rate might be lower.^22,23^ Data from Italy implicate that forearm-AVF are more frequently created if the nephrologist creates the access as opposed to a vascular surgeon.^24^ This indicates that local traditions, and physicians’ opinions play a role in the choice of access.

The aim of the study was to investigate how patient-specific factors as body size and comorbidities influenced the choice of haemodialysis access in a real-life setting.

## Method

### Data collection and observation time

In this retrospective cohort study, we included all patients registered in the Swedish Renal Registry (SNR) who received a haemodialysis access between 1 January 2013 and 31 December 2022. Registration in SNR is voluntary, and all patients have the right to withdraw their consent. Ethical approval for this study was obtained from the Swedish Ethical Review Authority (Dnr 2023-00124-01) and complied with the Declaration of Helsinki.

### Exclusion criteria

We excluded patients who solely received a non-tunnelled catheter.

### Baseline variables and comorbidities

Data regarding age, sex, cause of kidney failure, previous kidney replacement therapy (KRT), height and weight (after dialysis), were collected from SNR.

Data on comorbidities were extracted both from SNR and the National Patient Register (NPR). In the SNR, comorbidities are registered once, at start of first KRT. These are based on information from the treating nephrologist. The NPR carries data on diagnosis set by the practicing clinician for all hospitalizations and all specialist outpatient visits. Comorbidities were defined according to the presence of the following ICD 10-codes during the study period: Hypertension (I10- I15, O10.0), diabetes mellitus (E10-E11, E13-E14), ischaemic heart disease (I20-I25, Z95.1, Z95.5), cerebrovascular disease (I61-I66, I67.0, I67.2, I69.1, I69.3, I69.4, G45.0, G45.1, G45.2, G45.8, G45.9, G46), peripheral arterial disease (I73.9, I70.2), atrial fibrillation/flutter (I48), heart valve disease (I34-I37), heart failure (I50).

Types of diabetes mellitus were sometimes registered inconsistently in the same patient. We used the diagnosis with the most frequent registrations if the ratio was more than 2:1. If there was a more equal distribution of diagnosis codes, we classified diabetes mellitus as *unspecified*.

Vintage was defined as time from the start of first KRT. Body surface area (BSA) was calculated according to DuBois.

### Access

AV-accesses were grouped into four categories depending on location of artery, (1) forearm-AVF (from the radial or ulnar artery), (2) UAF (from the brachial artery), (3) AVG (in the arm) and (4) others (e.g. lower extremity). Patients who only received tunnelled CVC were recorded as ‘CVC-only’.

We ranked accesses in order of creation during the study period. Some patients had more than one access created the same day. Here, we considered CVC as first, forearm-AVF as second and UAF or AVG as last.

### Outcome

**Figure 1.**
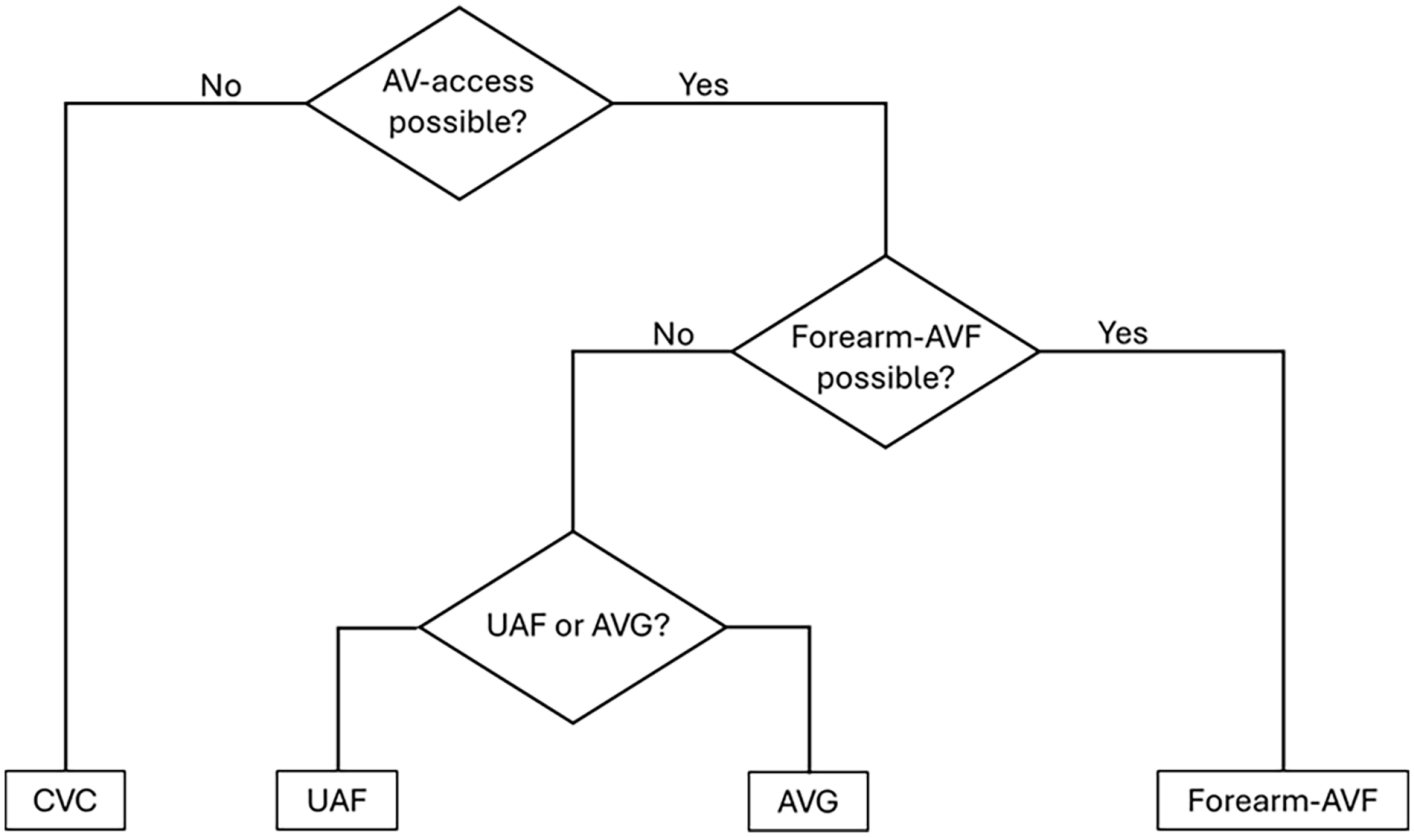
Clinical decision tree for the choice of first permanent haemodialysis access. CVC: central venous catheter. Arteriovenous (AV) accesses as forearm-arteriovenous fistula (forearm-AVF), upper-arm fistula (UAF), arteriovenous graft (AVG).

Outcomes were the changes in haemodialysis access over time and the patient-specific predictors contributing to the choice of first access during the study period. The analysis was performed at the different levels of a clinical decision tree; (1) AV-access versus CVC only, (2) forearm-AVF versus UAF/AVG and (3) UAF versus AVG (Figure 1).

### Statistical analysis

All statistical analyses were performed using IBM SPSS Statistics for Windows (Version 29.0.2.0 Armonk, NY: IBM Corp). Data were reported as mean, standard deviation (SD), median, inter quartile range (IQR) or frequencies (%) as appropriate. ANOVA was used for comparison between groups and change over time. Chi-square was used for nominal data and logistic regression for continuous variables. Results are reported as relative risk (95% CI) for nominal data and odds ratio (95% CI) for continuous variables. The statistical significance level was set at *p* < 0.05.

## Results

**Figure 2.**
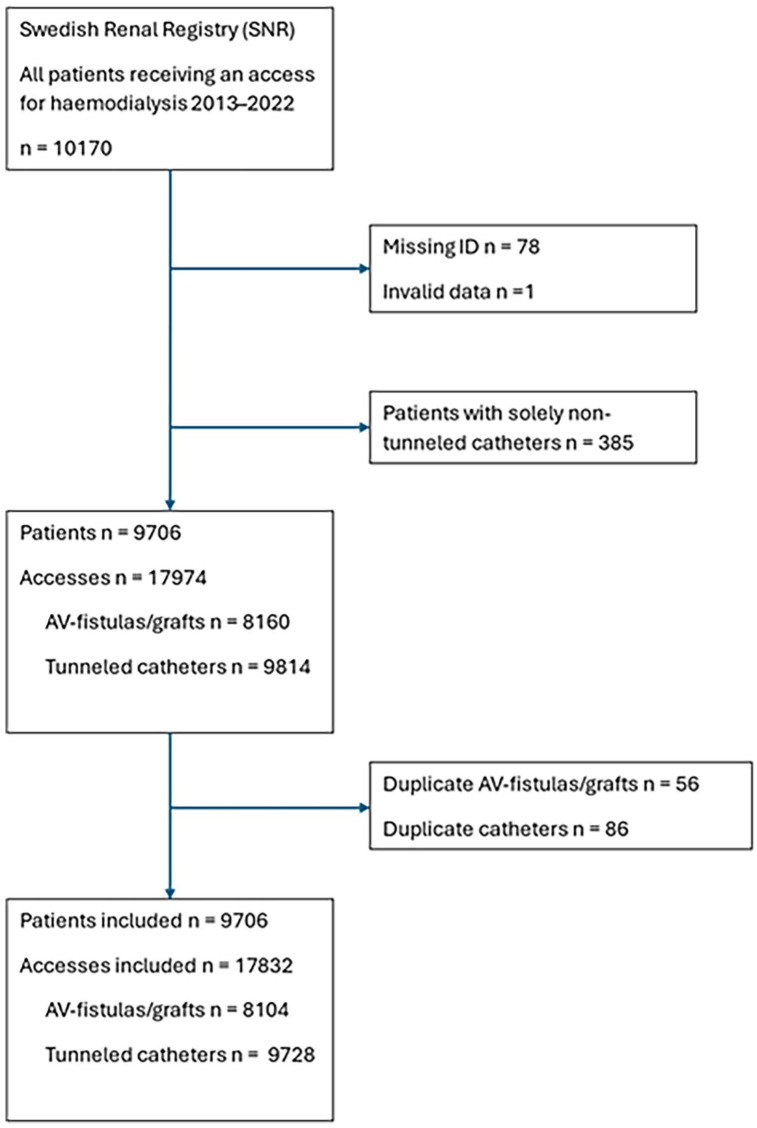
Study flowchart.

In the first dataset 10,170 patients were included. Of these, 78 patients were excluded due to unknown date of birth and sex. One patient was excluded due to incomplete treatment data. Finally, 385 patients were excluded since they only received a non-tunnelled catheter. In total, we included 9706 patients with 17,832 created haemodialysis accesses (Figure 2). Baseline characteristics are presented in Table 1.

**Table 1.** Baseline characteristics.

Age (years)	Mean	SD
At start of kidney replacement therapy	61.3	17.4
At first access during study period	64.0	15.4
Body metrics		
Weight (kg)	75.8	18.3
BMI (kg/m^2^)	26.0	5.7
Height (cm)	171	11
Body surface area (m^2^)	1.87	0.24
Sex	*n*	%
Men	6328	65.2
Women	3378	34.8
Primary kidney diagnosis		
Autosomal dominant polycystic kidney disease	685	7.1
Diabetes nephropathy	2422	36.0
Glomerulonephritis	1369	14.1
Hypertension	1569	16.2
Renovascular disease	78	0.8
Pyelonephritis	338	3.5
Other	2064	21.3
Unspecified	892	9.2
Missing	289	3.0
Comorbidities		
Hypertension	9203	94.8
Ischaemic heart disease	3859	39.8
Cerebrovascular disease	2211	22.5
Peripheral arterial disease	2052	21.1
Atrial fibrillation/flutter	3156	32.5
Heart valve disease	1314	13.5
Heart failure*	2145	22.1
Diabetes mellitus	4794	49.4
Type 1	718	7.4
Type 2	3751	38.6
Other specified forms	86	0.9
Unspecified	239	2.5
Previous KRT		
Any	4602	47.4
Transplantation	2674	27.5
Peritoneal dialysis	2562	26.4
Haemodialysis before study period	1387	14.3
First access**		
Catheter	5750	59.2
Forearm fistula	2490	25.7
Upper-arm fistula	918	9.5
Graft	511	5.3
Other	37	0.4
First AV-access**		
Forearm fistula	4124	62.9
Upper-arm fistula	1585	24.2
Graft	777	11.9
Other	68	1.0
Catheter only	3152	32.5

* Heart failure diagnosed before first access creation during study period.

** First access during study period. Potential accesses before study start are not accounted for.

**Figure 3.**
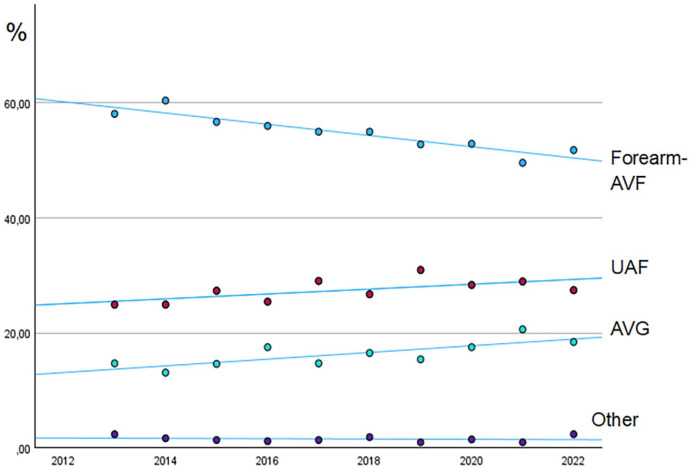
Proportion of created AV-accesses in Sweden 2013–2022. Forearm-AVF: forearm arteriovenous fistula; UAF: upper-arm fistula; AVG: arteriovenous graft.

A total of 4460 forearm-AVF (55.0%), 2211 (27.3%) UAF, 1310 (16.2%) AVG and 123 (1.5%) other AV-accesses were created during the study period and included in the analysis. Patients with fistulas from the ulnar artery represented 1% of patients with forearm-AVF. We found an increased proportion of UAF (*p* = 0.042) and AVG (*p* = 0.007) and a decreased proportion of forearm-AVF over the studied period (*p* < 0.001; Figure 3). Age did not change over the studied period (*p* = 0.377). The yearly number of accesses created was stable (mean 1797, SD 85).

At time of creation of ‘other’ accesses, patients were younger, had fewer comorbidities and more previous AV-accesses than the other groups. We considered them to represent special clinical cases and excluded them from further analysis.

More men than women got a forearm-AVF compared with all other groups (*p* < 0.001). There were significant differences in body size (length, weight, BMI, BSA) between access groups, with higher values in the forearm-AVF-group (*p* < 0.001 for all measurements). Patients with forearm-AVF had fewer comorbidities. Age did not differ between access groups. Vintage was shortest in the forearm-AVF-group and the longest in the AVG-group (*p* < 0.001). When excluding the CVC-only group, hypertension, heart valve disease and type 1 diabetes were no longer significant. All details are presented in Table 2.

**Table 2.** Patient characteristics at time of access creation.

Patients *n* = 9706	All accesses *n* = 17,709	CVC *n* = 9728	Forearm-AVF *n* = 4460	UAF *n* = 2211	AVG *n* = 1310	*p* ^a^	*p* ^b^
Sex (women)	36.5	38.0	29.0	40.2	44.0	<0.001	<0.001
Age (years)	63.8 (15.2)	63.8 (15.7)	63.7 (14.8)	64.0 (14.4)	64.1 (13.7)	0.803	0.613
Height (cm)	171 (10)	170 (11)	172 (10)	170 (11)	169 (10)	<0.001	<0.001
Weight (kg)	77.5 (19.0)	76.5 (19.0)	81.2 (19.0)	77.2 (18.6)	75.6 (18.8)	<0.001	<0.001
BMI (kg/m^2^)	26.7 (6.1)	26.5 (6.1)	27.4 (6.0)	26.5 (5.9)	26.5 (6.1)	<0.001	<0.001
BSA (m^2^)	1.88 (0.24)	1.87 (0.24)	1.92 (0.24)	1.87 (0.24)	1.85 (0.24)	<0.001	<0.001
Access number	1.8 (1.3)	1.8 (1.4)	1.6 (0.9)	2.0 (1.3)	2.3 (1.5)	<0.001	<0.001
Vintage (years)	3.2 (7.0)	3.7 (7.2)	1.7 (5.4)	3.6 (7.7)	4.5 (8.0)	<0.001	<0.001
Hypertension	83.1	84.3	81.4	81.0	83.0	<0.001	0.333
Cerebrovascular disease	13.2	14.4	10.2	13.9	13.4	<0.001	<0.001
Atrial fibrillation/flutter	6.2	6.4	5.7	6.5	6.0	0.375	0.435
Ischaemic heart disease	33.7	36.5	28.1	32.1	34.4	<0.001	<0.001
Peripheral arterial disease	11.4	13.2	7.5	11.4	11.0	<0.001	<0.001
Heart valve disease	6.2	7.0	4.9	6.2	5.3	<0.001	0.087
Heart failure	23.5	25.8	19.9	19.9	25.4	<0.001	<0.001
Diabetes mellitus, all	42.8	43.3	40.5	43.5	45.3	0.002	0.003
Diabetes mellitus, type 1	6.6	7.5	5.0	6.1	5.6	<0.001	0.217
Diabetes mellitus, type 2	34.2	33.7	33.7	35.3	37.5	0.030	0.034

CVC: central venous catheter; Forearm-AVF: forearm arteriovenous fistula; UAF: upper-arm fistula; AVG: arteriovenous graft; BMI: body mass index; BSA: body surface area.

Data are reported as mean (SD) or percentage.

a ANOVA comparing all groups. ^b^ANOVA with CVC-only excluded.

Predictors for the type of the first access are presented in Table 3. Forest plots for RR are presented in Supplemental Material.

**Table 3.** Patient-related factors affecting the choice of first access.

	AV access vs CVC only^a^			Forearm-AVF vs UAF or AVG^b^			UAF vs AVG^c^		
Factor	OR	CI	*p*	OR	CI	*p*	OR	CI	*p*
Age (per year)	1.004	(1.002–1.007)	0.002	1.002	(0.999–1.006)	0.229	1.000	(0.994–1.006)	0.994
Height (cm)	0.991	(0.987–0.996)	<0.001	0.976	(0.970–0.981)	<0.001	0.989	(0.981–0.998)	0.012
Weight (kg)	0.982	(0.979–0.985)	<0.001	0.982	(0.979–0.985)	<0.001	0.991	(0.986–0.997)	0.002
BMI (kg/m^2^)	0.945	(0.935–0.954)	<0.001	0.967	(0.957–0.977)	<0.001	0.986	(0.969–1.002)	0.089
BSA (m^2^)	0.292	(0.233–0.367)	<0.001	0.216	(0.168–0.278)	<0.001	0.454	(0.299–0.691)	<0.001
Vintage (per year)	1.020	(1.013–1.026)	<0.001	1.054	(1.044–1.062)	<0.001	1.010	(0.999–1.021)	0.076
	RR	CI	*p*	RR	CI	*p*	RR	CI	*p*
Sex (female)	1.144	(1.097–1.213)	<0.001	1.425	(1.337–1.518)	<0.001	0.915	(0.863–0.970)	0.003
Hypertension	0.698	(0.631–0.771)	<0.001	0.700	(0.619–0.791)	<0.001	1.057	(0.927–1.204)	0.386
Ischaemic heart disease	1.002	(0.945–1.063)	0.936	1.021	(0.956–1.090)	0.534	1.021	(0.964–1.081)	0.484
Cerebrovascular disease	0.975	(0.910–1.045)	0.469	1.042	(0.967–1.124)	0.282	1.014	(0.949–1.082)	0.690
Peripheral arterial disease	1.079	(1.008–1.155)	0.031	1.233	(1.147–1.326)	<0.001	1.012	(0.948–1.080)	0.720
Atrial fibrillation/flutter	0.980	(0.922–1.042)	0.520	1.016	(0.949–1.088)	0.651	1.020	(0.961–1.082)	0.520
Heart valve disease	0.974	(0.894–1.060)	0.538	1.088	(0.995–1.189)	0.069	1.033	(0.956–1.115)	0.424
Heart failure	1.053	(0.994–1.116)	0.079	1.042	(0.977–1.112)	0.212	0.948	(0.895–1.005)	0.071
Diabetes (all)	0.900	(0.850–0.953)	<0.001	1.008	(0.945–1.074)	0.819	0.980	(0.926–1.037)	0.487
Diabetes, type 1	1.283	(1.169–1.408)	<0.001	1.153	(1.024–1.297)	0.023	1.024	(0.922–1.136)	0.668
Diabetes, type 2	0.816	(0.767–0.868)	<0.001	0.969	(0.908–1.035)	0.353	0.989	(0.934–1.048)	0.710
Previous peritoneal dialysis	1.476	(1.393–1.564)	<0.001	1.045	(0.969–1.127)	0.256	1.020	(0.955–1.089)	0.560
Previous transplantation	0.954	(0.894–1.018)	0.151	1.029	(0.959–1.105)	0.425	0.989	(0.928–1.053)	0.726
Haemodialysis before study	1.237	(1.150–1.331)	<0.001	1.813	(1.695–1.939)	<0.001	0.811	(0.746–0.881)	<0.001

CVC: central venous catheter; Forearm-AVF: forearm arteriovenous fistula; UAF: upper-arm fistula; AVG: arteriovenous graft; BMI: body mass index; BSA: body surface area; OR: odds ratio; RR: relative risk.

a OR/RR >1 favours CVC only. ^b^OR/RR >1 favours UAF or AVG. ^c^OR/RR >1 favours AVG.

Peritoneal dialysis (PD) before access creation increased the probability of CVC-only. The median time on CVC was 221 days (IQR 323) for those who had PD before and 228 days (IQR 369) for those who had not had PD (*p* = 0.225).

## Discussion

Forearm-AVF are still the primary choice 2013–2022 in Sweden. The creation of UAF is however increasing. This is in line with older data from Sweden and with published international data from DOPPS 2012–2014.^19,25^ The increase of UAF in DOPPS was most pronounced in the USA, amounting to 68%. In contrast, the use of Forearm-AVF accounts for 95% of vascular access modalities in Japan.^26^ In contrast to DOPPS where the prevalence of AVG remained at a stable level, we saw an increase.^19^ The age in the patient population did not change during the same period, indicating that other factors affected the choice of access.

Previous epidemiological access studies mainly focused on patency and complications. In those studies, patients with AVG or CVC were often older and had more comorbidities than patients with fistulas and had worse outcomes.^12,13,27,28^ In our cohort, when studying all created accesses, patients had more comorbidities at the time of access creation for CVC and AVG. Additionally, body size parameters differed between access groups. We could however not see any age differences. Ultimately, 32.5% of the patients remained on CVC throughout the study. This is in line with previously published data from Italy, reporting 34.5%.^24^ In a recently published study from Austria, CVC use varied widely between centres. In addition to vessel status and comorbidities, patient preference and organizational barriers were identified as underlying reasons.^29^

We further analysed the choice of the first permanent access. To our knowledge this is the first study focusing on patient-specific factors influencing this choice.

One of the important factors is body size. Smaller body size, regardless of mode of measurement, increased the risk to be dialysed via CVC and decreased the probability of receiving a forearm-AVF. Smaller body size also increased the probability of receiving an UAF over an AVG. Previous data indicate that vessel size is the main factor affecting fistula maturation.^22,23^ This might be an indication that vessel size is an important factor involved in choosing an access for the individual patient.

The same pattern was seen for female sex. While decreased primary patency in women has been reported,^22^ women’s vessel diameter does not differ much from men’s.^30^ Internationally, the proportion of forearm-AVF in women differs significantly.^19,31^ Therefore, other factors such as physician practice patterns may influence the choice of access.^30^

Comorbidities other than PAD and diabetes mellitus did not seem to influence the choice of access. A previous study demonstrated a decreased need for revisions and shorter time to first use in patients with diabetes mellitus receiving AVG compared to fistulas.^28^ This might be an indication that vessel quality is another factor involved in choosing an access for the individual patient. Patients with hypertension less often ended up with CVC, and they had a higher chance of receiving a forearm-AVF when choosing between AV-accesses. The 5.2% of the study population without hypertension were younger and therefore these results are difficult to generalize. There was an indication that heart failure may increase the probability of CVC and AVG, but it did not reach statistical significance.

Previous haemodialysis influenced the choice of access on all decision levels, increasing the probability of CVC only, other AV-accesses over forearm-AVF and UAF over AVG. For these patients, the vascular opportunity to create a forearm-AVF might already have been exhausted. Vintage had a higher impact than age, which strengthens this theory.

Previous PD increased the probability to receive a CVC maybe due to a plan to return to PD or a perceived short time to transplantation. However, total days on CVC did not differ between patients who previously had PD and those who had not.

When forearm-AVF is not an option and the choice is between UAF and AVG, female sex, small body size and previous HD were the only significant factors, all favouring UAF. This emphasizes the potential importance of the vessel size and quality as a factor in decision-making. The fact that AVG had a significantly higher number of previous accesses indicates that clinicians favour UAF-creation before resorting to AVG.

In this study we focused on patient-specific factors influencing the choice of access. Previous studies and both international and national data suggest large variation between countries^19^ as well as between centres.^24,32^ We did not analyse centre-specific data and it is likely that there is an added effect of centre- or clinician’s preference. Previous data also suggests that patients are more likely to receive a forearm-AVF if the nephrologist is involved in the decision making.^24^ Dedicated programmes can reduce the use of UAF.^33,34^

Under the study period, relevant international guidelines were updated^1^ and usually, there is a delay in their implementation into clinical practice. Applying a vessel-centred rather than a patient-centred approach, might be in transition.

When looking at survival, patency and complications for different kinds of accesses retrospectively, patient-related factors affecting the choice of access might bias the outcome. Both patient-related factors and the access itself could explain higher risks for heart failure, death and other complications. Ultimately, how to choose *the right access for the right patient at the right time for the right reasons* will need randomized trials.

### Strength and limitations

SNR is a registry with high national coverage and high validity.^35^ Still, the results are based on the data entered. As an observational retrospective study, we cannot exclude residual confounders impacting access choice. Diagnoses from the NPR are recorded from health services in specialist care but usually not set at the time of access creation. There might have been a delay when prevalent comorbidities were recorded. Comorbidities in the SNR are registered at first start of KRT and are considered precise. Combining these two sources, SNR and NPR, we think that the validity is as good as possible with retrospective data. Another limitation is the lack of data on accesses created before the study period. We did not have access to measurements of vessel size or quality. Our conclusions are based on the knowledge that the factors and comorbidities identified directly impact vessel size and/or quality. We did not have information about patient preference or socioeconomic status. In Sweden, the cost of medical treatment is capped at a maximum of approximately 110–140 USD/Euro per year. Any costs exceeding this amount are covered and do not have to be paid by the patient.

## Conclusions

UAF and AVG are increasingly created as haemodialysis accesses. Forearm-AVF still seems to be the primary choice. Cardiac comorbidities or age do not seem to affect the decision. Small body size, female sex, PAD, diabetes mellitus and previous haemodialysis treatment are the most important factors affecting the choice of access. This might indicate that the clinician’s approach when choosing access is vessel-centred rather than patient-centred.

## Supplemental Material

sj-pdf-1-jva-10.1177_11297298251357632 – Supplemental material for Patient-related factors influencing the choice of haemodialysis access in Sweden Supplemental material, sj-pdf-1-jva-10.1177_11297298251357632 for Patient-related factors influencing the choice of haemodialysis access in Sweden by Emelie Laveborn, Ellinor Bergdahl, Ulrika Hahn Lundström, Bernd Stegmayr and Michael Ott in The Journal of Vascular Access
