# Supplementary material for: Patient-related factors influencing the choice of haemodialysis access in Sweden
Source: J Vasc Access. 2025 Jul 23;27(2):607–15. doi: 10.1177/11297298251357632 (PMC12920699; doi:10.1177/11297298251357632)

Patient-related factors influencing the choice of haemodialysis access in Sweden

Supplement

Relative risk of receiving arteriovenous access vs central venous catheter only

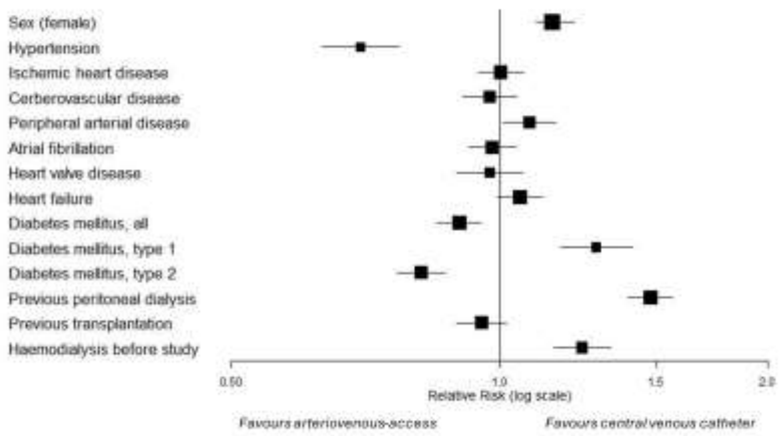

Relative risk of reciving forearm fistula vs upperarm fistula or arteriovenousgraft

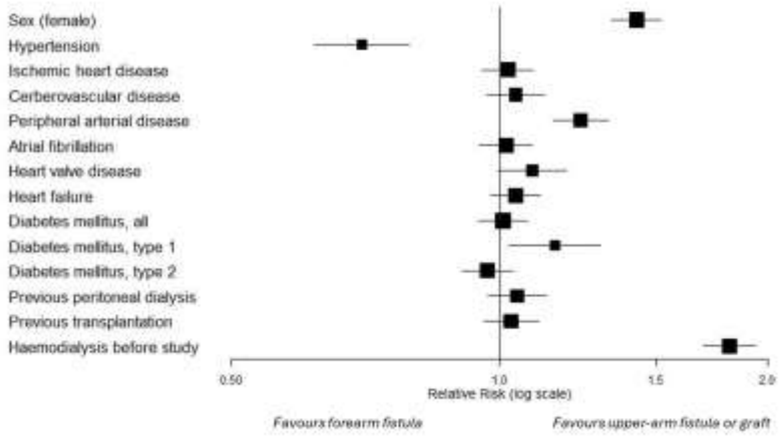

Relative risk of receiving upper-arm fistula vs arteriovenous graft

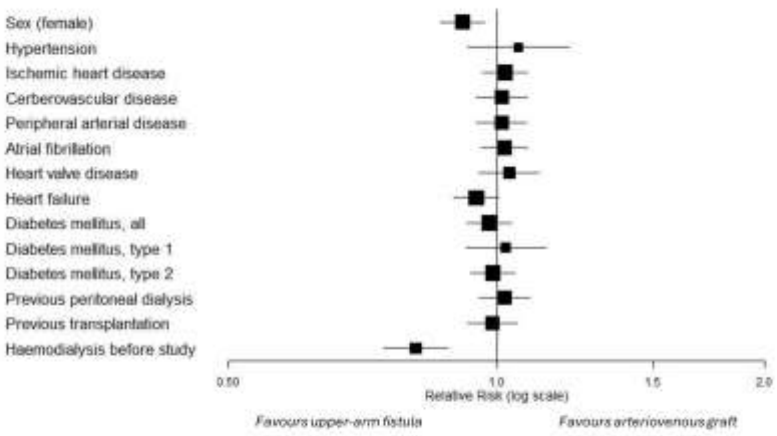

Supplement: sj-pdf-1-jva-10.1177_11297298251357632 – Supplemental material for Patient-related factors influencing the choice of haemodialysis access in Sweden [file sj-pdf-1-jva-10.1177_11297298251357632.pdf]
